# Combination of stem cell-derived secretome from human exfoliated deciduous teeth with Yemeni Sidr honey on cell viability and migration: an in vitro study

**DOI:** 10.1038/s41405-024-00197-5

**Published:** 2024-03-13

**Authors:** Mona Abdulrahman Abdullah Al-Hadi

**Affiliations:** 1 Faculty of Dentistry, Airlangga University, Surabaya, Indonesia; 2 Faculty of Dentistry, University of Science and Technology, Sana’a, Yemen

**Keywords:** Dentistry, Dental materials

## Abstract

**Introduction:**

Bone diseases have a profound global impact, especially when the body’s innate regenerative capacity falls short in the face of extensive damage. Stem cells from human exfoliated deciduous teeth (SHEDs), discovered in 2003, offer a promising solution for tissue repair, as they self-renew naturally and are easily obtainable. Mesenchymal stem cells (MSCs), including SHEDs, are believed to promote tissue regeneration by releasing growth factors, collectively known as the secretome.

**Aims:**

This study explored the potential of combining SHED-derived secretome with Yemeni Sidr honey to improve osteoblast and fibroblast cell viability and migration.

**Materials and methods:**

The experiment involved treating cell cultures of two types of rat cell lines - 7F2 osteoblast and BHK-21 fibroblast immortalized cells - with SHED-derived secretome and Yemeni Sidr honey. After the treatment, cell viability was measured using the MTT assay, which calculates OD at 590 nm. Additionally, the scratch assay was conducted to evaluate cell migration, and ImageJ software was used for data processing.

**Results:**

The findings indicated that combining SHED-derived secretome and Yemeni Sidr honey enhanced osteoblast and fibroblast cell viability and migration. Furthermore, the study highlighted the difference in the stimulative potential of SHED-derived secretome, Yemeni Sidr honey, and their combination, on the viability and migration of the cultured cells.

**Conclusion:**

The research concludes that combining SHED-derived secretome with Yemeni Sidr honey has the potential to promote cell viability and migration in in-vitro settings. The synergistic application of these substances has been found to be more effective -when combined in a dose-dependent manner- than their counterparts. Overall, the current study serves as a foundation for further investigations to establish if the explored substance has any useful clinical applications.

## Introduction

Numerous bone diseases have a negative impact on a person’s quality of life and cost the global healthcare system finances [1]. On July 14, 2022, the WHO released its most recent update, which estimates that 1.71 billion people worldwide suffer from musculoskeletal conditions, including bone diseases. People who have musculoskeletal conditions and related functional limitations are becoming more and more prevalent as a result of population growth and aging.

Reconstruction of bone defects caused by fractures, tumors, infections, or congenital diseases is a significant challenge in oral and maxillofacial surgery and orthopedics. Despite the fact that bones are able to repair and regenerate when they are damaged by large lesions, the healing process is unsuccessful, and the damage does not heal on its own [2]. In the reconstruction of bone defects larger than the critical size, current therapies have primarily focused on the use of grafts and bone substitutes, which have been extensively utilized yet have certain drawbacks and limitations [2, 3]. These include a scarcity of graft tissue, donor-site pain and morbidity, and the requirement for two surgical procedures. Furthermore, there are concerns about the recipient’s immune system rejecting the donor tissue and facilitating disease transmission [4]. In order to adequately regenerate and restore these defects, this has prompted the search for new therapeutic options [5, 6]. This could pave the way for regenerative medicine, which aims to repair or replace damaged cells and tissues in organs to restore normal function. Bone regeneration and remodeling have been intensively investigated in recent decades with the goal of developing new therapeutic approaches for fracture healing, treatment of bone defects, and implant osteointegration. New regenerative approaches have been created, including guided tissue regeneration, tissue engineering, application of stem cells and growth factors, high-tech fixation devices, and customized implants [7–12].

Stem cells from human exfoliated deciduous teeth (SHEDs) were first discovered in 2003 [13], are self-renewing MSCs (Mesenchymal Stem Cells) found in the dental pulp’s perivascular niche. Because teeth naturally fall out throughout childhood, obtaining SHED is simple and convenient, with little or no trauma and fewer ethical concerns. Furthermore, deciduous tooth dental pulp is present from birth and lasts until permanent teeth erupt. This period is distinguished by the maintenance of an active niche rich in stem cells, which are strong and proliferative and have not yet been heavily influenced by the cumulative effects of genetic and environmental factors [14]. For all of these reasons, SHEDs are one of the most popular sources of stem cells for biomedical engineering [15]. Furthermore, numerous in vitro and in vivo studies have shown that SHEDs have several advantages over dental pulp stem cells from permanent teeth (DPSCs), including higher rates of proliferation and differentiation [16, 17], higher rates of osteogenic and adipogenic differentiation [18], abundant supply of extracellular matrix and growth factors [16], and increased proliferation in culture under unfavorable environments (hypoxia, high glucose, low serum) [19].

It is currently thought that MSCs’ main mechanism of action in tissue regeneration and repair is through the release of growth factors, cytokines, and extracellular matrix molecules, which have a paracrine effect on host cells, modulating endogenous cell migration, angiogenesis, and cell differentiation, and inducing the repair and regeneration of injured tissues [20–23]. Secreted factors are known as a secretome and can be found in the medium used to cultivate mesenchymal stem cells, known as a conditioned medium (MSC-CM) [22, 24]. In contrast to engrafting stem/progenitor cells, cell-free secretome approach could avoid the risks of tumorgenicity, antigenicity, host rejection, and infection associated with stem cell-based therapies, making it a safer and more practical source for regenerative bioactive molecules [25].

A natural product like honey is well known for its compositional diversity and medicinal properties such as anti-inflammatory activity, wound healing efficacy, and nutritional competence. Honey’s multifaceted nature appeals to regenerative medicine researchers [26]. Due to its exceptional antimicrobial efficacy and tissue-regenerative capabilities, honey has been utilized as a treatment modality since ancient times [27]. Proinflammatory cytokines were demonstrated to be less active when honey was incorporated into materials used in tissue regeneration and engineering because of its ability to reduce inflammation, which aided in beneficial healing. Additionally, in-vitro and in-vivo scientific studies revealed that their administration markedly boosted angiogenesis, reepithelialization, and granulation tissue formation [28].

One of the best authentic kinds of honey, which is regarded as one of the best original honey produced in the Arab world, is Sidr Honey [29]. The Arabs consider Yemeni Mountain Sidr honey to be superior to the more expensive Manuka honey from New Zealand, which has received more attention in the West. There is still no definitive answer in the publications as to what distinguishes definite kinds of honey from others. Today’s market is rife with adulterated artificial honey that masquerades as natural [30]. Yemeni Sidr honey is regarded as the finest and highest quality honey in the world, owing to the fact that Yemen’s climatic conditions and environment are ideal for honey production. Furthermore, the bee constructs its hive without human intervention, conserving its natural specifications and essential live enzymatic components [29]. All in all, Yemeni Sidr honey is the best quality and most appreciated type of honey available all around the world. It helps to promote health, healing, and immune system support in a number of different ways. Due to its abundance of essential elements and minerals, it is a potent antibiotic and antibacterial agent, with strong anti-inflammatory, antipyretic, and analgesic properties [29, 31].

Accordingly, the researcher draws several conclusions from the earlier studies that serve as the basis for this study’s motivation, which are as follows:

1. Since the majority of research focuses on manuka honey [32–36], no studies have examined the role of Sidr honey in bone healing and regeneration. This research will be the first to shed light on this issue.
2. Due to the complexity of the paracrine activity of the MSCs secretomes, the majority of SHED-Secretome-related studies are still conducted in vitro or in vivo rather than clinically, necessitating more research before this type of treatment can be more reliably applied in actual clinical settings.
3. Combining stem cell-derived secretome with honey is a new approach that has not been researched before. As a result, the current study will be the initiation of further investigations to determine whether this substance has any useful clinical applications.

## Materials and methods

### Study design

The study used an experimental randomized post-test-only control group design, and the protocol was approved by the Faculty of Dentistry Research Ethics Commission, Airlangga University, Surabaya, Indonesia, with ethical clearance No. 462/HRECC.FODM/V/2023 (approval date: May 5, 2023). The research was carried out on cultures of 7F2 osteoblast cells and BHK-21 fibroblast cells exposed to Stem Cell-Derived Secretome from Human Exfoliated Teeth and Yemeni Sidr honey, which were divided into seven groups, including: honey at three different concentrations (0.02%, 0.50%, 0.75%), secretome, and the combination of both. Each undergoing the treatment on four occasions, accompanied by the controls (control cell and control media). The research findings were evaluated using cell viability and migration tests.

### Human SHED-derived secretome isolation and preparation

SHED was obtained from the Research Center at the Faculty of Dental Medicine, Universitas Airlangga, derived from primary teeth that met the criteria for being free of caries, had no roots that had undergone resorption, and had a vital and intact pulp. The pulp was isolated by extirpation under aseptic conditions, then cut into small pieces (0.5 mm), rinsed with phosphate-buffered saline (PBS), and then soaked for an hour at 37 °C in a solution containing type I collagenase and dispase. The cell suspension was put in a culture dish with Dulbecco’s modified Eagle medium (DMEM) and fetal bovine serum (FBS), and then 5 mm L-glutamine, 100 μ/ml penicillin-G, 100 μ/ml streptomycin, and 100 μ/ml kanamycin were added. The dish is then incubated at 37 °C. Then DMEM without serum was used to replace the culture medium. At intervals of four days, the media were incubated for 48 h at 37 °C in a humid environment with 5% CO_2_. Unbound cells were removed from the culture medium by switching it. For up to four portions, culture jars and cells were kept. Cellular debris was cleaned out using PBS. When transferring cells to a larger culture dish, trypsin-EDTA 0.05% was used to release the cells. SHED cells at the fourth passage are then ready for the secretome retrieval step once the cell has reached 70–80% confluence [37].

Aspirate the 70–80% confluent SHED culture medium. Place the conical tube with the culture medium inside, then centrifuge. The supernatant was taken, deposited, and the pellets and supernatant were then discarded and added to a dialysis tube with ties at both ends. The tube containing the supernatant was placed in a glass beaker that already had PBS solution, and it was incubated at 4 °C above a plate magnetic stirrer until the medium color dried up. The supernatant from the dialysis tube was taken out, put in a conical tube, and centrifuged once more to create pellets at the bottom of the tube. The supernatant was filtered without pellets using a 0.22 μL filter and stored in tubes at −20 °C [38].

### Preparation of the combination (Secretome/Honey)

SHED-derived Secretome /Honey combination was prepared by mixing secretome solution of 200 μL, and honey (50%) with three different concentrations: 15 μL (0.75%), 10 μL (0.50%), and 4 μL (0.02%). The solution of each group was labeled in microtubes for further use.

Bioactive components found in both SHEDs secretome and honey make their combination significant. The diverse array of these compounds from both sources holds great importance as follows; SHEDs Secretome contains anti-inflammatory cytokines and GFs such as IL-10, TGF-, VEGF, and FGF-2. The GF is expected to boost bone regeneration by increasing anti-inflammatory macrophage (M2) polarization, angiogenesis, differentiation, and proliferation of osteoblast cells. IL-10 found in the SHEDs Secretome promotes the polarization of the M2 phenotype by activating signaling pathways such as signal transducer and activator of transcription-3 (STAT3). TNF- activates Th2 cells via the TNFR2 receptor. Th2 expresses IL-4 to activate polarization, and the M1 phenotype transitions to M2 via the STAT6 signaling pathway. M2 cells produce the anti-inflammatory cytokine IL-10, as well as the growth factors VEGF and FGF-2. IL-10 suppresses osteoclastogenesis by inhibiting TNF- and IL-1, as well as RANKL by stimulating OPG increase by OB. VEGF and FGF-2 can stimulate angiogenesis, which is required for osteoblast cell migration, differentiation, and proliferation. FGF-2, in conjunction with TGF-, can promote proliferation, inhibit apoptosis, recruit OB progenitors to bone formation sites, and promote OB progenitors’ differentiation into preosteoblasts (pre-OB). Pre-OB expresses BMP-2 to activate RUNX2 and Osx transcription factor signaling pathways SMADs 1/5/8. RUNX2 plays an important role in pre-OB differentiation. Pre-OB secretes Osx, which regulates gene expression in osteoblasts by inhibiting NF-κB signaling which results in increasing the OPG/RANKL expression ratio, inhibiting osteoclastogenesis, upregulating ALP, and the OCN, which plays a role in increasing calcium-phosphate deposition in the matrix mineralization process bone in bone regeneration.

Honey contains phenolic and flavonoid compounds, which are primarily responsible for its antioxidant properties. Free radical scavenging, hydrogen donation, singlet oxygen quenching, and metal ion chelation are some of the antioxidant activities of phenolic compounds. Honey’s exogenous nonenzymatic antioxidants can support endogenous antioxidant enzymes, and both enzymatic and enzymatic antioxidant components in honey may exert their effects at various cellular levels. Tyrosine kinases, protein kinase C (PKC), and MAPKs are just a few of the intracellular signaling cascades that polyphenols in honey can activate. In addition, honey has anti-inflammatory properties, including the ability to reduce levels of proinflammatory mediators like NO, prostaglandin E2 (PGE2), TNF-, and IL-6. Furthermore, honey flavonoid extracts displayed anti-inflammatory activity by reducing NF-B translocation to the nucleus, preventing IB degradation, and suppressing COX-2. Additionally, the p65 acetylation-dependent activation of NF-Bs and the production of inflammatory markers were inhibited by garlic acid, a class of phenolic acids present in honey. These bioactive substances work in concert to support honey’s overall anti-inflammatory properties.

By combining SHEDs-derived secretome and honey, it is possible to inhibit the formation of osteoclasts while promoting bone formation by fibroblasts and osteoblasts. This process can help to preserve and improve bone density and strength, ultimately leading to better bone healing and regeneration. (Supplemental Material 1)

### Preparation of osteoblast and fibroblast cell cultures

7F2 osteoblast and BHK-21 fibroblast Immortalized rat cell lines were obtained from the Research Center at the Faculty of Dental Medicine, Universitas Airlangga. The preparation was conducted in laminar flow. The 7F2 osteoblast cell culture was housed in a roux bottle with DMEM (Dulbecco’s Modified Eagle Medium) and 10% Fetal bovine serum (FBS). It was then incubated for 24 h at 37 °C. The DMEM media and FBS 10% growth media were then removed from the roux bottle. The osteoblast cell culture in the roux bottle was then rinsed with PBS. For 5 min, trypsin 0.05% was used to eliminate cells adhered to the bottom of the roux container. The liberated cell culture was subsequently given a new DMEM medium containing 10% FBS. Based on the number of samples and controls, cell cultures that have been replenished with new media were divided into 96 wells of the Tissue Culture Plate. Cells were cultured on well plates with growth media for the treatment groups and the cell control group. The 8-well plates were only for growth medium, with each well containing 1 × 10^4^ osteoblast cells and 100 μL DMEM. The plates were then incubated for 24 h at 37 °C with 5% CO_2_. A similar method was used to cultivate BHK-21 fibroblast cells [39].

### Preparation of cell viability assay (MTT assay)

Based on a modification to the technique described by [40] a 3-(4,5-Dimethyl-2-thiazolyl)-2,5-diphenyl-2H- tetrazolium bromide MTT assay was carried out to evaluate the cell viability. Before the treatment materials were applied, the osteoblast cells were seeded in a 96-well plate at a density of 1 × 10^4^ cells/well in 100 μL medium. They were then given 24 h to adhere at 37 °C in a CO_2_ incubator. The culture medium was changed to a brand-new medium after 24 h of incubation. Each well was then filled with 10 μL of the MTT working solution, and the plate was incubated for 4 h at 37 °C in a CO_2_ incubator. Following the aspiration of the medium, the formed formazan crystals were solubilized by adding 50 μL of DMSO to each well and incubating for 30 min at 37 °C in a CO_2_ incubator. The intensity of the dissolved formazan crystals (purple color) was finally measured at 590 nm using an ELISA plate reader. The percentage of viable cells had a direct correlation with the amount of absorption (color intensity generated). The fibroblast cells went through the same process. The percentage of osteoblast and fibroblast cells that remain viable after being exposed to treatment materials was calculated using the following equation [41]:

$${{{{{\rm{Cell}}}}}}\,{{{{{\rm{viability}}}}}} \% =\frac{{{{{{\rm{treatment}}}}}}\,{{{{{\rm{group}}}}}}-{{{{{\rm{blank}}}}}}\,{{{{{\rm{group}}}}}}}{{{{{{\rm{Control}}}}}}\,{{{{{\rm{group}}}}}}-{{{{{\rm{blank}}}}}}\,{{{{{\rm{group}}}}}}}\times 100$$

- Cell viability %: Percentage of the number of cells alive after treatment
- Treatment group: Value of each sample after testing
- Blank group : Values on the average of each control medium
- Control group : Values on the average control cell

### Preparation of the cell migration assay (Scratch assay)

To carry out the scratch assay, cells were seeded onto the entire surface of a 35 mm culture µ-dish and left to adhere and spread on the substrate for 6 h or overnight at 37 °C and 5% CO_2_. A wound was created in the cell monolayer by carefully scratching it with a sterile P-200 pipette tip in a straight line at the center of the plate. The tip was pushed continuously with a consistent size in each well to ensure consistency. To remove floating cells and debris, the plate was washed twice with 1 ml of sterile 1X PBS before adding 2 ml of suitable media for the assay. The 1X PBS and medium were pre-warmed at 37 °C to avoid cell detachment during the wash. Imaging was done using a phase-contrast scanning confocal microscope with a CO_2_ microscope cage incubator at 37 °C and 5% CO_2_, and precise placements and focal planes were determined to eliminate overlapping fields. Multiple photographs were taken across the 0–24–48 h 10X objective, and four fields were chosen from each wound for each sample. Data processing was done using open-source software (ImageJ) [42].

### Statical analysis

The statistical analysis was carried out using SPSS version 25. The data was presented as mean ± standard deviation (X ± SD). The normality test was performed using the Shapiro-Wilk test, while the homogeneity test was conducted using Levene’s test. Additionally, a One-Way ANOVA test and Post hoc (HSD Tukey) test were performed with a significance level of 0.05 [43].

## Results

### Cell viability test results on osteoblast and fibroblast cell cultures

The study involved testing the viability of Human SHED-Derived Secretome, Yemeni Sidr honey, and their combination on 7F2 Osteoblasts and BHK-21 Fibroblasts using the MTT assay. The ELISA reader was used to read the optical density values, which indicate the percentages of living cells, cell death, and the absorbance value of the sample color. The color is produced by cell metabolism, which converts the MTT reagent into a purplish-blue formazan product. The more saturated the color, the higher the absorbance value. The absorbance values were divided into 7 treatment groups, each of which received the treatment four times, along with the controls (control cell and control media). After obtaining the absorbance values for each well, the percentage of living cells and the mean of each concentration were determined. The following formula was used to conduct the calculations:

$$	{{{{{\rm{Percentage}}}}}}\,{{{{{\rm{of}}}}}}\,{{{{{\rm{Living}}}}}}\,{{{{{\rm{Cells}}}}}}\\ 	=\frac{({{{{{\rm{Treat}}}}}}.\,{{{{{\rm{Absorbance}}}}}}-{{{{{\rm{Control}}}}}}\,{{{{{\rm{Media}}}}}}\,{{{{{\rm{Absorbance}}}}}})}{({{{{{\rm{Control}}}}}}\,{{{{{\rm{Cell}}}}}}\,{{{{{\rm{Absorbance}}}}}}-{{{{{\rm{Control}}}}}}\,{{{{{\rm{Media}}}}}}\,{{{{{\rm{Absorbance}}}}}})}\times 100 \% \,$$

### Cell viability test results on 7F2 Osteoblast cell culture

**Fig. 1:**
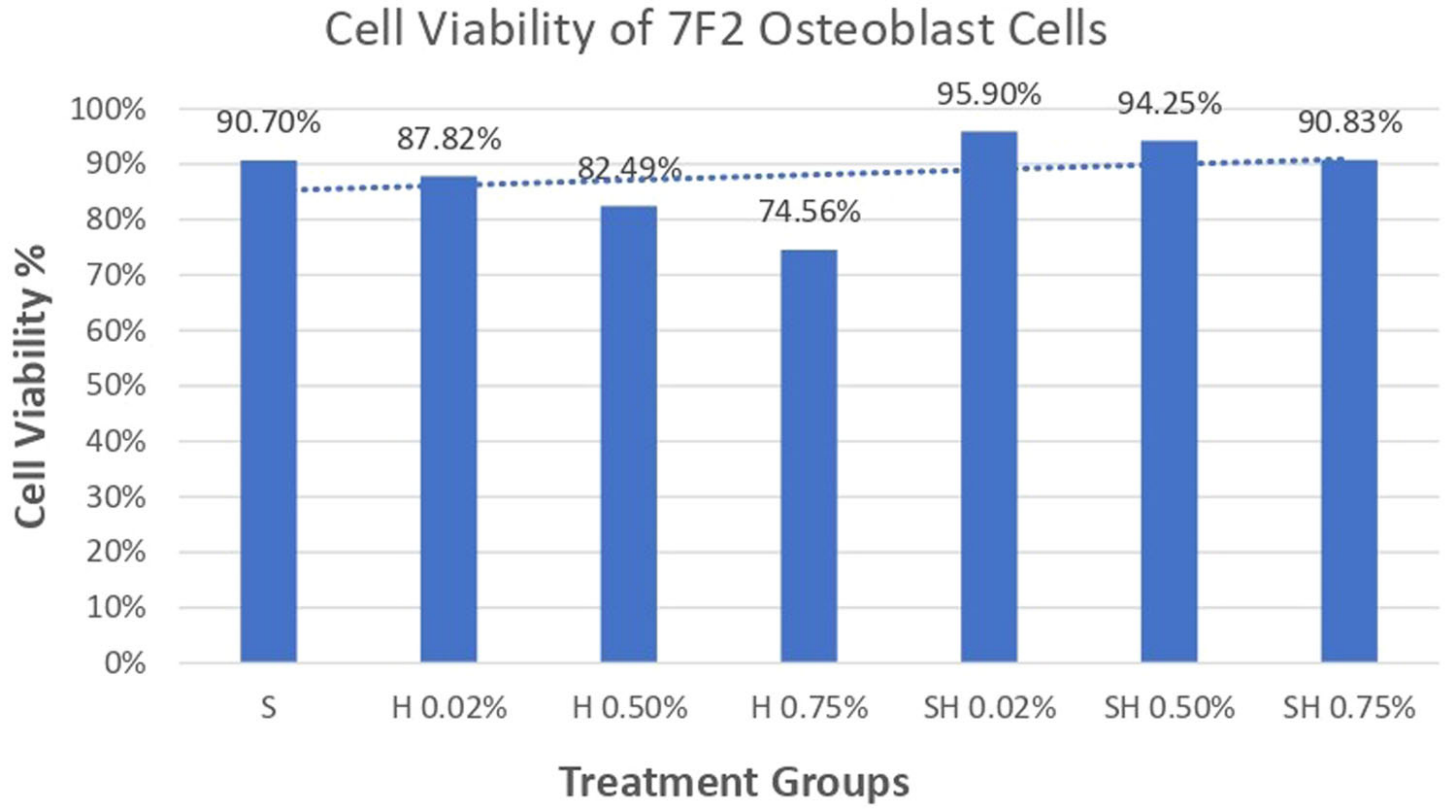
Cell Viability Percentage of 7F2 Osteoblast Cells.

**Fig. 2:**
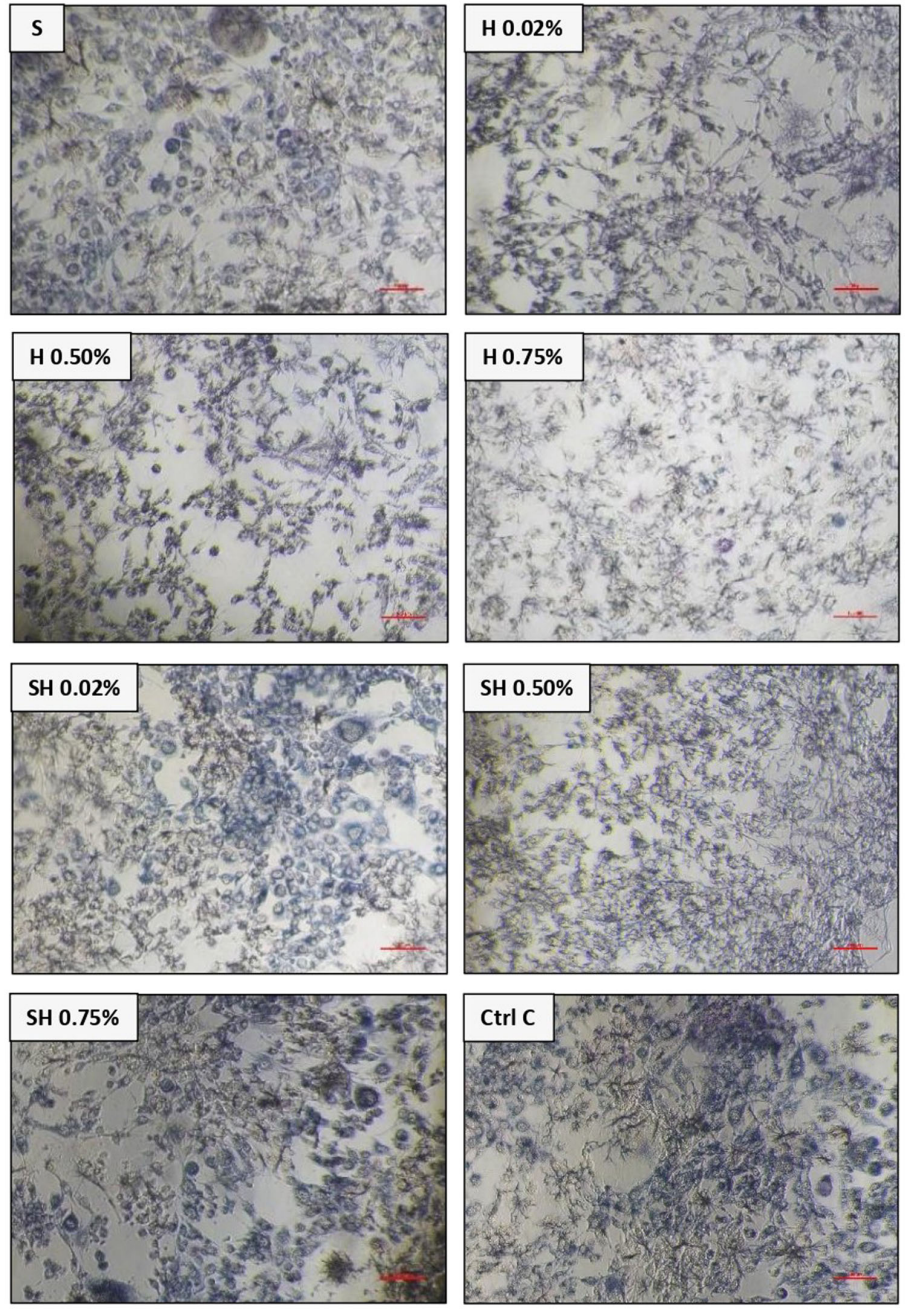
7F2 osteoblast cells under a light microscope at 10× magnification among treatment groups [Secretome, Honey (0.02%,0.50%,0.75%), Secretome & Honey (0.02%,0.50%,0.75%)] and control cell group.

Absorbance results of 7F2 osteoblast cells after administration of Human SHED-Derived Secretome, Yemeni Sidr honey, and the combination of both through ELISA reader obtained the following results in Table 1. Table 2 shows the percentage of viable cells as well as the mean and standard deviation in different treatment groups as well as the control cell. According to Fig. 1, the treatment group Secretome & Honey (0.02%) had the highest proportion of surviving cells (94.25%) when compared to the control cell. Meanwhile, the lowest number of osteoblast cells was seen in the Yemeni Sidr Honey (0.75%) treatment group (74.56%). The percentage of live cells indicates the sample’s cell viability value. The increase in the number of osteoblast cells is proven by comparing the mean survival percentages for the control cell group at 100% (Fig. 2). Furthermore, the graph demonstrates that following the highest concentration of living cells, there was a modest decline in the viability percentage of the cells in the Secretome & Honey treatment group (0.50%). While for the treatment group of Secretome alone, the percentage is 90.70%, the honey treatment groups at concentrations of 0.02% and 0.50% were 87.82% and 82.49%, respectively. Based on the Shapiro-Wilk test results, the osteoblast cell data group appeared to be normally distributed (*p* > 0.05). However, the homogeneity test on the groups, as indicated by Levene’s test results, showed a significance value of *p* < 0.05, suggesting that there is a notable difference in data variance. Additionally, the One-way ANOVA test results for the osteoblast cell data demonstrated a *p* value of 0.000, indicating a significant difference overall. To identify any significant differences between groups, a Post hoc (Tukey HSD) test was conducted and the results are presented in Table 3.

**Table 1:** Absorbance values of osteoblast cells.

Replica	Treatment Groups							Control Cell	Control Media
	S	H 0.02%	H 0.50%	H 0.75%	SH 0.02%	SH 0.50%	SH 0.75%		
1.	0.335	0.218	0.19	0.212	0.221	0.186	0.125	0.328	0.069
2.	0.247	0.17	0.235	0.287	0.164	0.285	0.19	0.199	0.069
3.	0.138	0.319	0.228	0.186	0.319	0.31	0.173	0.318	0.072
4.	0.221	0.213	0.228	0.138	0.275	0.186	0.454	0.164	0.068
Average	0.24	0.23	0.22	0.21	0.24	0.24	0.24	0.25	0.07

*S* Secretome, *H* Honey, *SH* Secretome & Honey.

**Table 2:** The cell viability values of 7F2 osteoblast cells.

	Groups	Mean OD ± SD	Percentage of living cells average (%)
1.	Control cell	0.25 ± 0.08	100%
2.	Human SHEDs Secretome	0.24 ± 0.08	90.70%
3.	Yemeni Sidr Honey (0.02%)	0.23 ± 0.06	87.82%
4.	Yemeni Sidr Honey (0.50%)	0.22 ± 0.02	82.49%
5.	Yemeni Sidr Honey (0.75%)	0.21 ± 0.06	74.56%
6.	Secretome & Honey (0.02%)	0.24 ± 0.07	95.90%
7.	Secretome & Honey (0.50%)	0.24 ± 0.06	94.25%
8.	Secretome & Honey (0.75%)	0.24 ± 0.15	90.83%

**Table 3:**
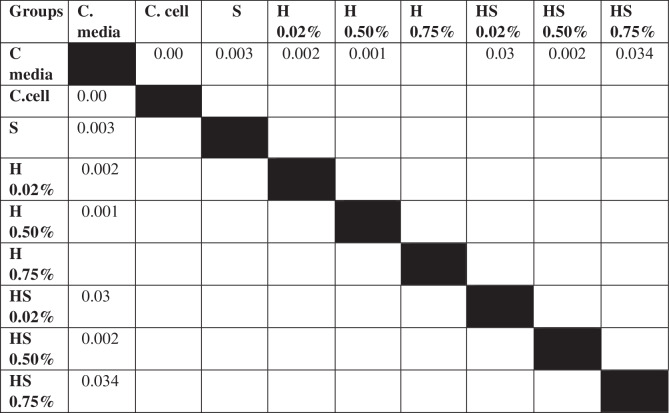
The *P* value of the Tukey HSD test for 7F2 osteoblast cell viability between each study group.

### Cell viability test results on BHK-21 Fibroblast cell culture

**Fig. 3:**
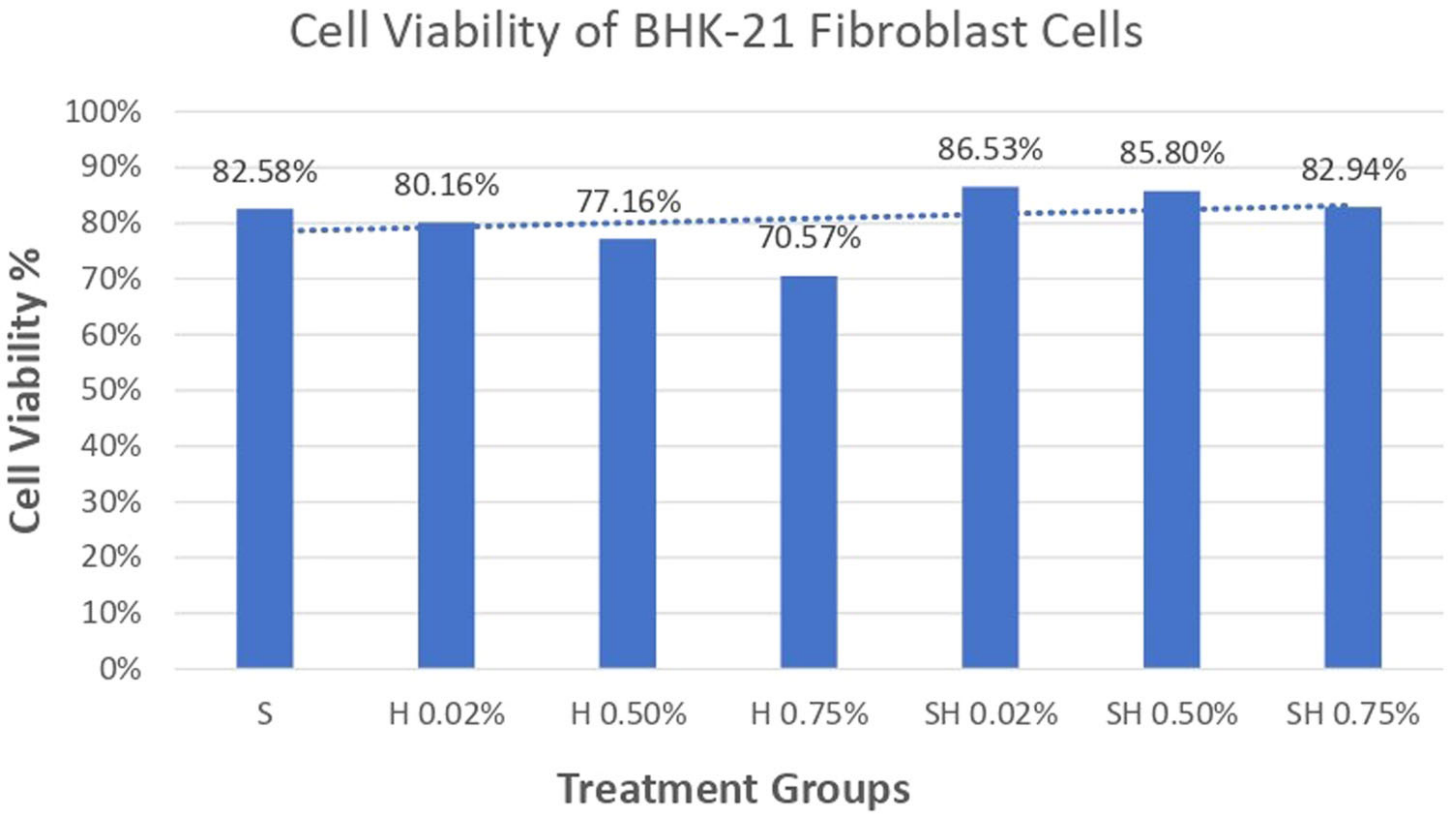
Cell Viability Percentage of BHK-21 Fibroblast Cells.

**Fig. 4:**
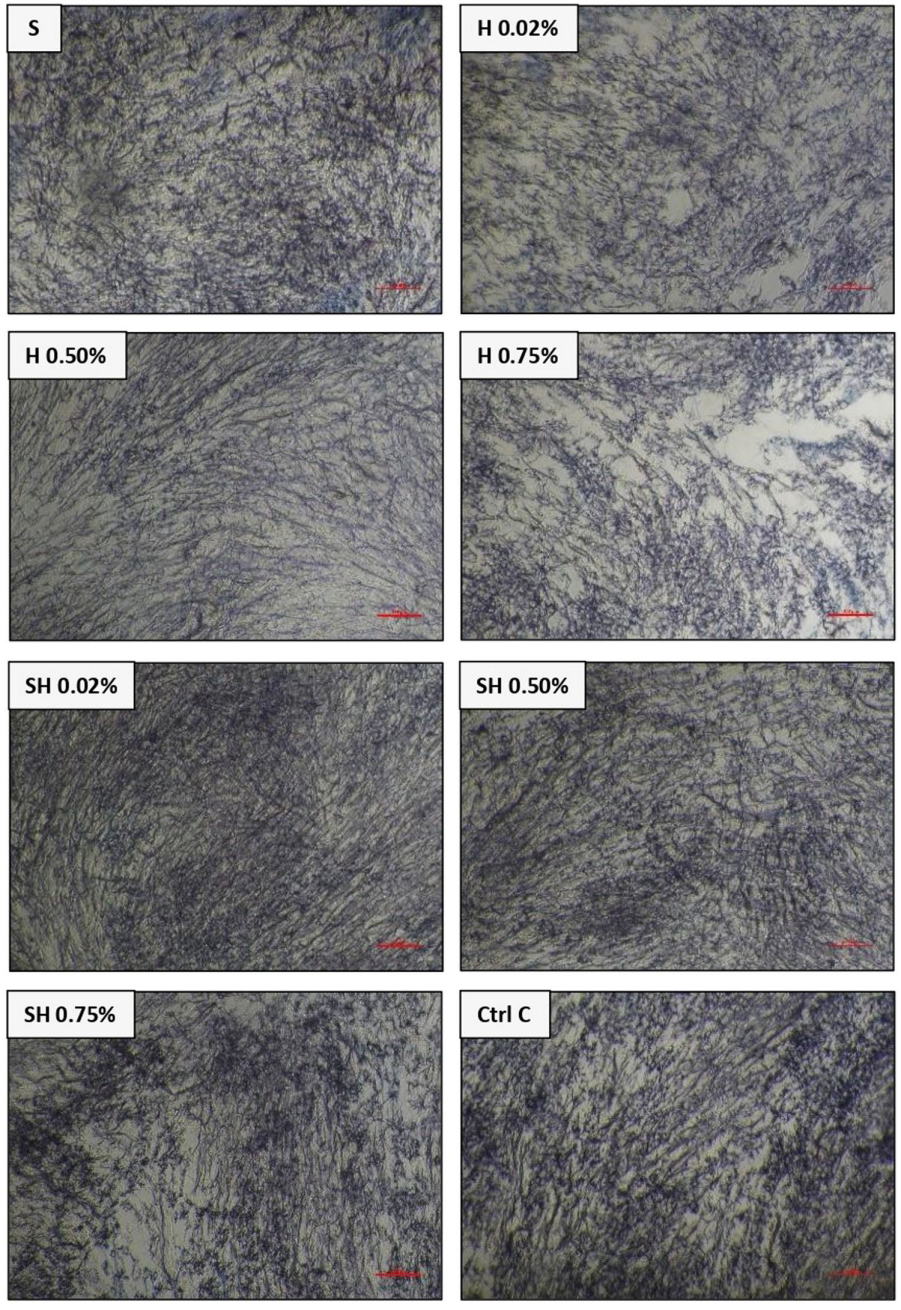
BHK-21 fibroblast cells under a light microscope at 10× magnification among treatment groups [Secretome, Honey (0.02%,0.50%,0.75%), Secretome & Honey (0.02%,0.50%,0.75%)] and control cell group.

Absorbance results of BHK-21 fibroblast cells after administration of Human SHED-Derived Secretome, Yemeni Sidr honey, and the combination of both through ELISA reader obtained the following results in Table 4. Table 5 shows the percentage of viable cells as well as the mean and standard deviation in different treatment groups as well as the control cell. According to Fig. 3, the treatment group Secretome & Honey (0.02%) had the highest proportion of surviving cells (86.53%) when compared to the control cell. Meanwhile, the lowest number of osteoblast cells was seen in the Yemeni Sidr Honey (0.75%) treatment group (70.57%). The percentage of live cells indicates the sample’s cell viability value. The increase in the number of osteoblast cells is proven by comparing the mean survival percentages for the control cell group at 100% (Fig. 4). Furthermore, the graph demonstrates that following the highest concentration of living cells, there was a modest decline in the viability percentage of the cells in the Secretome & Honey treatment group (0.50%). While for the treatment group of Secretome alone, the percentage is 82.58%, the honey treatment groups at concentrations of 0.02% and 0.50% were 80.16% and 77.16%, respectively. According to the Shapiro-Wilk test results, the fibroblast cell data group appears to be normally distributed (*p* > 0.05). However, the results of Levene’s test indicate that the significance value of the fibroblast cell homogeneity test on the groups is *p* < 0.05, which suggests that the data variance is significantly different. Furthermore, the One-way ANOVA test revealed a *p* value of 0.009, indicating that there is a notable difference overall. To determine if there are any significant differences between groups, a Post hoc (Tukey HSD) test was conducted, and the results are presented in Table 6.

**Table 4:** Absorbance values of fibroblast cells.

Replica	Treatment Groups							Control Cell	Control Media
	S	H 0.02%	H 0.50%	H 0.75%	SH 0.02%	SH 0.50%	SH 0.75%		
1.	0.389	0.429	0.362	0.38	0.319	0.377	0.432	0.462	0.054
2.	0.569	0.292	0.342	0.346	0.485	0.418	0.472	0.525	0.068
3.	0.28	0.389	0.339	0.328	0.36	0.359	0.136	0.38	0.061
4.	0.132	0.227	0.253	0.152	0.26	0.26	0.335	0.241	0.059
Average	0.34	0.33	0.32	0.30	0.36	0.35	0.34	0.40	0.06

**Table 5:** The cell viability values of fibroblast cells.

	Groups	Mean OD ± SD	Percentage of living cells average (%)
1.	Control cell	0.40 ± 0.12	100%
2.	Human SHEDs Secretome	0.34 ± 0.18	82.58%
3.	Yemeni Sidr Honey (0.02%)	0.33 ± 0.09	80.16%
4.	Yemeni Sidr Honey (0.50%)	0.32 ± 0.05	77.16%
5.	Yemeni Sidr Honey (0.75%)	0.30 ± 0.10	70.57%
6.	Secretome & Honey (0.02%)	0.36 ± 0.09	86.53%
7.	Secretome & Honey (0.50%)	0.35 ± 0.07	85.80%
8.	Secretome & Honey (0.75%)	0.34 ± 0.15	82.94%

**Table 6:**
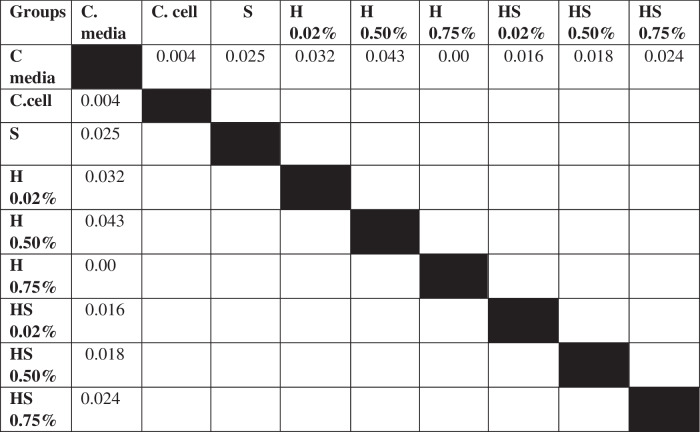
The *P* value of the Tukey HSD test for BHK-21 fibroblast cell viability between each study group.

### Cell migration test results on osteoblast and fibroblast cell cultures

**Fig. 5:**
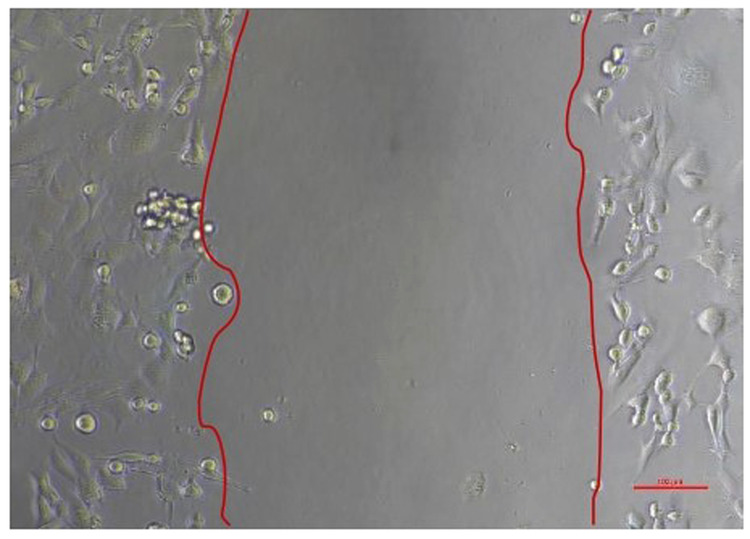
Scratch length of 7F2 osteoblast cells under light microscope at 10× magnification.

**Fig. 6:**
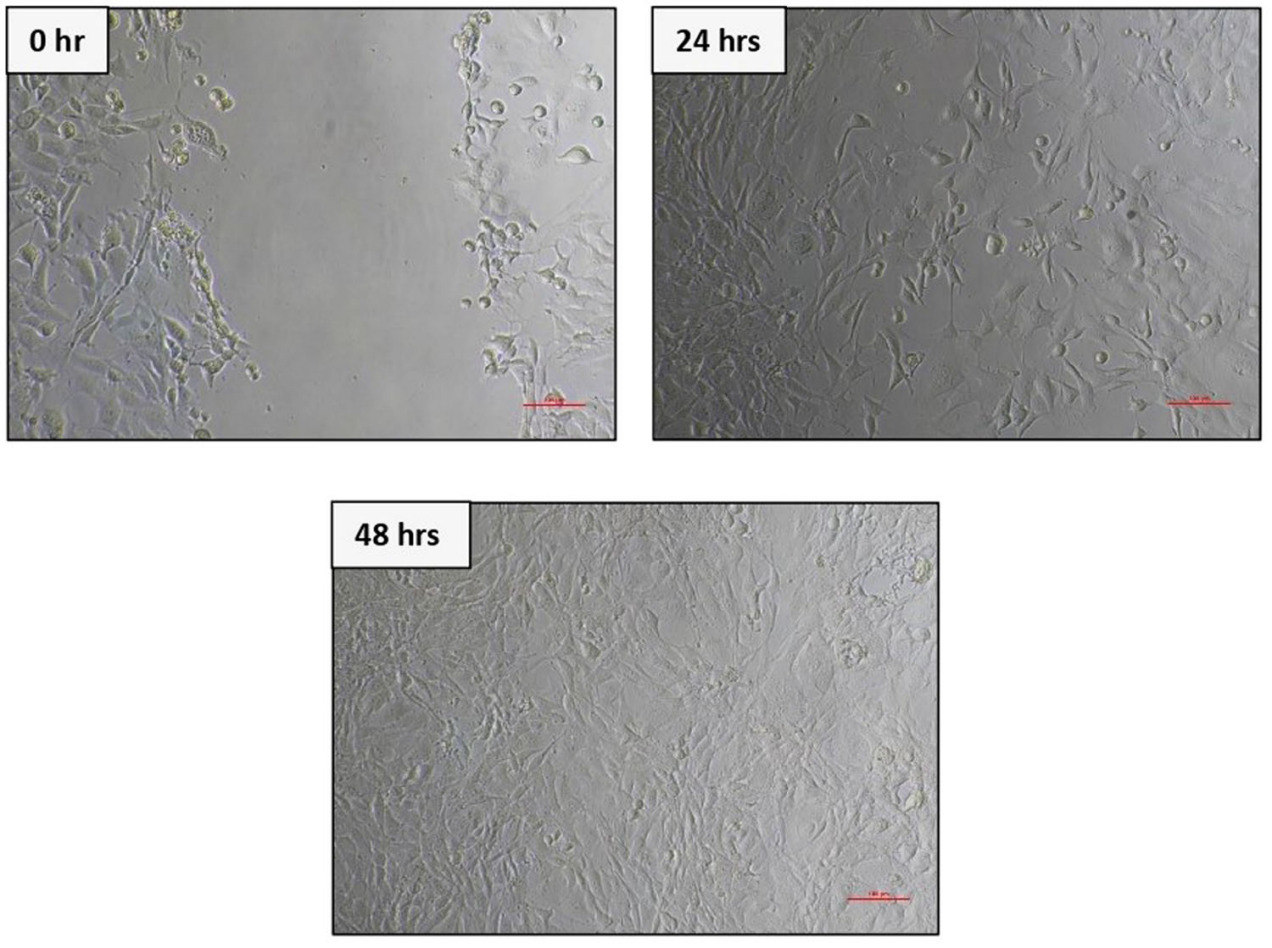
7F2 osteoblast cell migration under light microscope at 10× magnification at 3 different times (0 h, 24 h, 48 h).

**Fig. 7:**
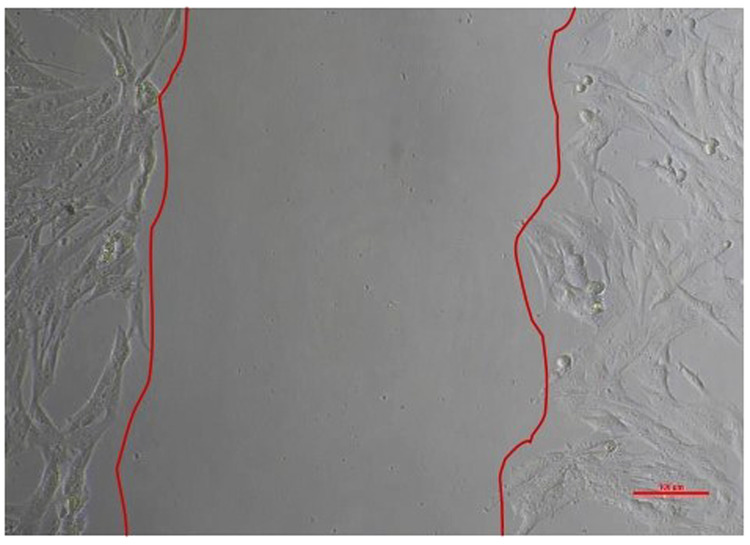
Scratch length of BHK-21 fibroblast cells under light microscope at 10× magnification.

**Fig. 8:**
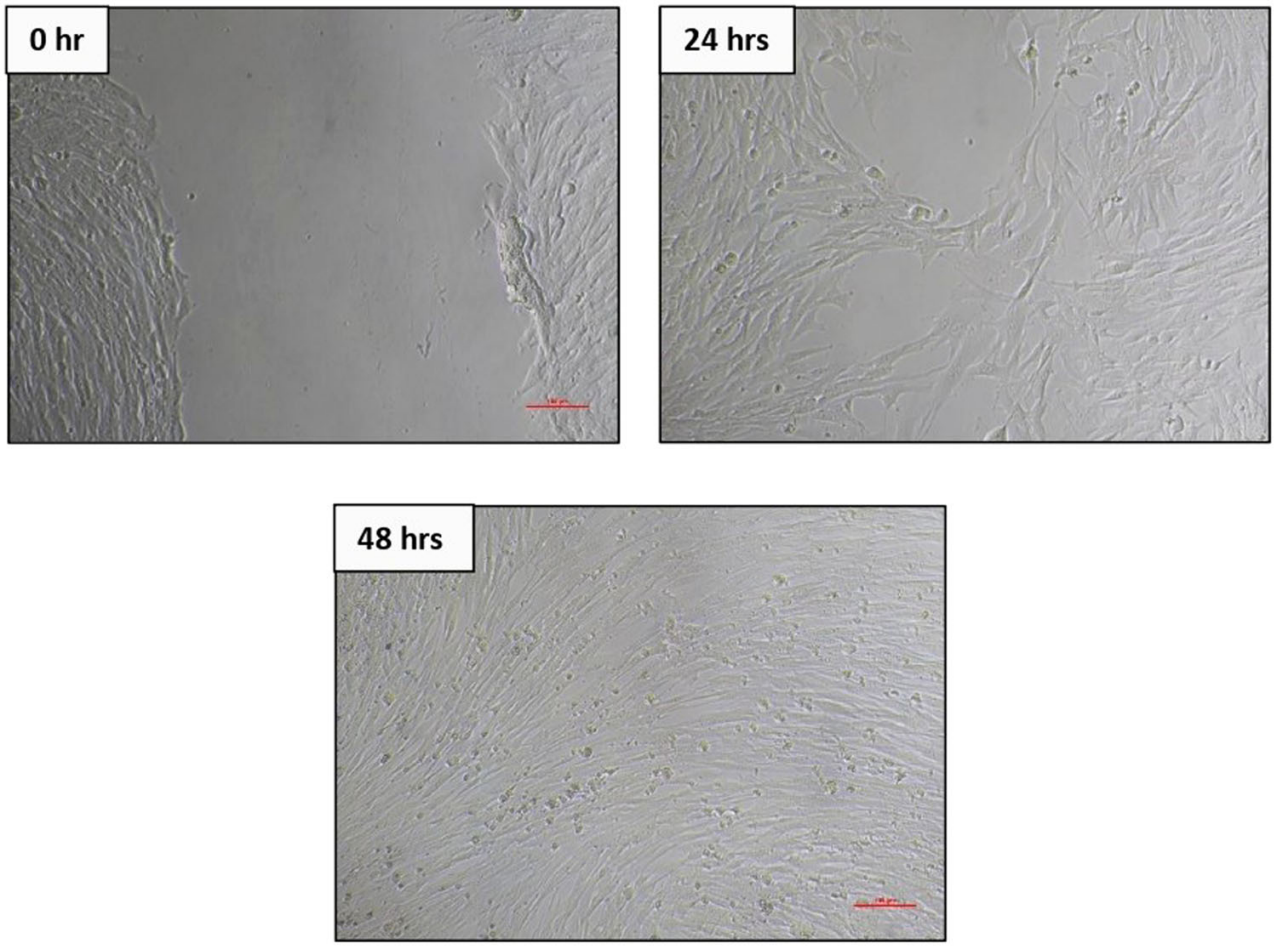
BHK-21 fibroblast cell migration under light microscope at 10× magnification at 3 different times (0 h, 24 h, 48 h).

The results of a cell migration test conducted on Human SHED-Derived Secretome, Yemeni Sidr honey, and the combination of the two on 7F2 Osteoblasts and BHK-21 Fibroblasts were analyzed using Open-source software (ImageJ). The length between the two sides of the scratch in images taken at initial (0 h), (24 h), and end time (48 h) was measured to determine cell front velocity Figs. 5, 6, 7, 8. Four sections of each image were measured between the closest spots on both sides of the wound. The more decreased the scratch length, the higher cell migration results. The values were then categorized into 7 treatment groups at 3 different times, each undergoing treatment with accompanying controls (cell and media).

### Cell migration test results on 7F2 Osteoblast cell culture

The results of cell migration for 7F2 osteoblast cells were obtained after the administration of Human SHED-Derived Secretome, Yemeni Sidr honey, and the combination of both, processed through Open-source software (ImageJ). These results are shown in Table 7 which displays the mean and standard deviation values for each treatment group, as well as the control cell, at three different time intervals. Figure 6 depicts the 7F2 osteoblast cell migration under a light microscope at 10× magnification. The Shapiro-Wilk test was conducted on the osteoblast cells group, which showed that the data were normally distributed (*p* > 0.05). Levene’s test results indicated that the data variance is significantly different, with a significance value of *p* < 0.05. The One-way ANOVA test results for osteoblast cells data are *p* = [0.000 (0 h), 0.002 (24 h), (48 h)0.002], indicating that there is a significant overall difference. The Post hoc (Tukey HSD) test was conducted to identify any significant differences between groups, which are detailed in Tables 8, 9, 10.

**Table 7:** The cell migration values of 7F2 osteoblast cells.

Mean ± SD	Treatment Groups (7F2 Osteoblast Cell Migration)							Control Cell
Time	S	H 0.02%	H0.50%	H 0.75%	SH 0.02%	SH 0.50%	SH 0.75%	
0 h	2241.37 ± 172.54	1857.09 ± 273.72	2190.09 ± 5.6407	2514.19 ± 49.857	2503.93 ± 80.222	2315.9 ± 203.01	2426.67 ± 133.88	2398.63 ± 197.88
24 h	1388.03 ± 366.51	862.22 ± 354.34	1145.98 ± 259.77	1107.01 ± 363.49	480 ± 113.55	1173.33 ± 119.24	990.09 ± 229.86	1338.8 ± 230.29
48 h	294.7 ± 139.11	324.79 ± 112.80	336.41 ± 143.67	547.01 ± 134.79	235.21 ± 76.87	525.13 ± 143.25	382.22 ± 49.90	215.38 ± 82.82

**Table 8:**
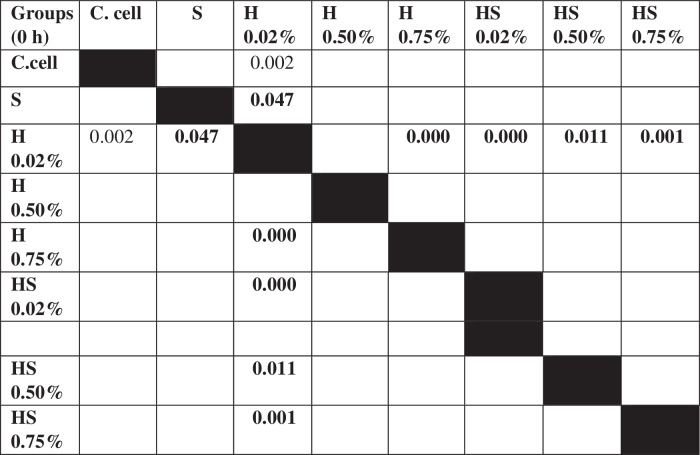
The *P* value of the Tukey HSD test for 7F2 osteoblast cell migration between each study group (0 h).

**Table 9:**
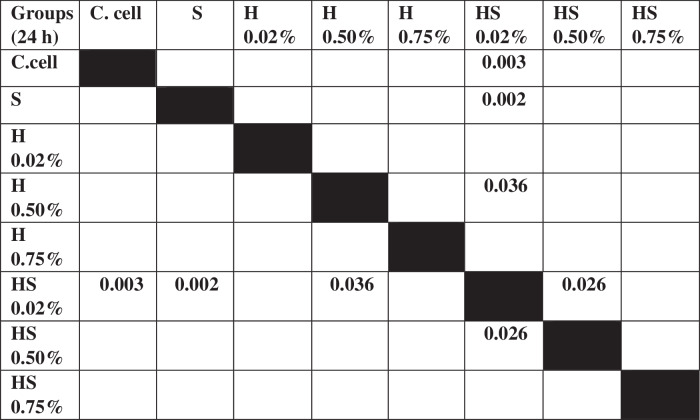
The *P* value of the Tukey HSD test for 7F2 osteoblast cell migration between each study group (24 h).

**Table 10:**
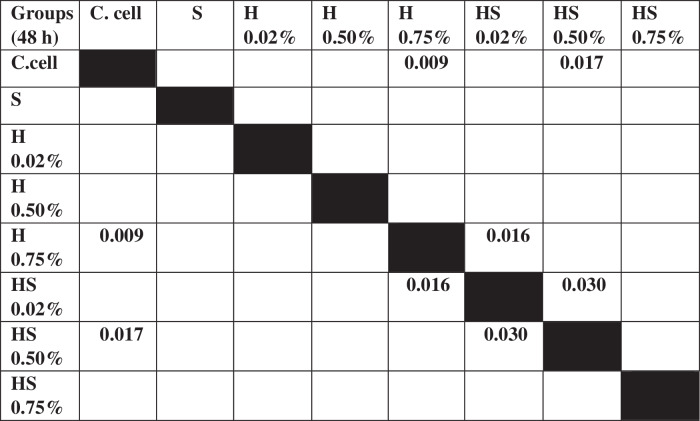
The *P* value of the Tukey HSD test for 7F2 osteoblast cell migration between each study group (48 h).

### Cell migration test results on BHK-21 Fibroblast cell culture

The results of cell migration in BHK-21 fibroblast cells after the administration of Human SHED-Derived Secretome, Yemeni Sidr honey, and a combination of both processed through Open-source software (ImageJ) were recorded in Table 11. The table shows the mean and standard deviation for each treatment group, as well as the control cell, at three different times. Figure 8 demonstrates BHK-21 fibroblast cell migration under the light microscope at 10× magnification. The Shapiro-Wilk test results on the fibroblast cells group showed that the data were normally distributed (*p* > 0.05). Levene’s test results showed that the significance value of the fibroblast cell homogeneity test on the groups has *p* < 0.05. Therefore, it can be interpreted that the data variance is significantly different. The One-way ANOVA test results for fibroblast cells data are *p* = [0.004(0 h), 0.001(24 h), 0.000(48 h)], indicating that there is a significant difference overall. The Post hoc (Tukey HSD) test was carried out to find out any significant differences between groups, which can be found in Tables 12, 13, 14.

**Table 11:** The cell migration values of BHK-21 fibroblast cells.

Mean ± SD	Treatment Groups (BHK-21 Fibroblast Cell Migration)							Control Cell
Time	S	H 0.02%	H 0.50%	H 0.75%	SH 0.02%	SH 0.50%	SH 0.75%	
0 h	2172.54 ± 115.85	2389.74 ± 89.98	1217.78 ± 875.93	2380.85 ±279.90	1610.26 ± 53.53	1813.33 ± 218.88	2449.23 ± 68.63	2369.23 ± 137.70
24 h	854.7 ± 109.53	1015.38 ± 319.50	1098.12 ± 189.93	1733.33 ± 308.81	649.57 ± 174.19	1152.82 ± 410.56	1069.4 ± 293.45	892.31 ± 192.48
48 h	221.54 ± 111.05	308.38 ± 95.25	464.27 ± 151.40	1032.48 ± 87.88	145.64 ± 49.85	234.53 ± 59.68	476.58 ± 242.97	279.66 ± 50.65

**Table 12:**
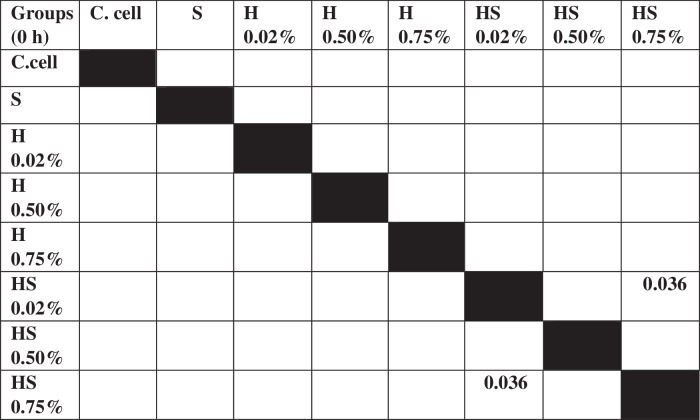
The *P* value of the Tukey HSD test for BHK-21 fibroblast cell migration between each study group (0 h).

**Table 13:**
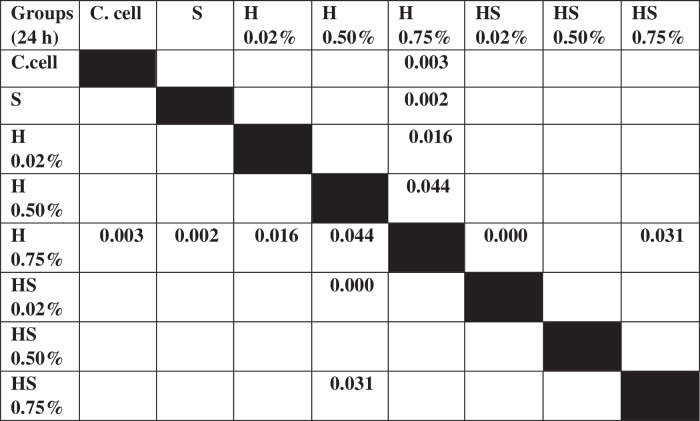
The *P* value of the Tukey HSD test for BHK-21 fibroblast cell migration between each study group (24 h).

**Table 14:**
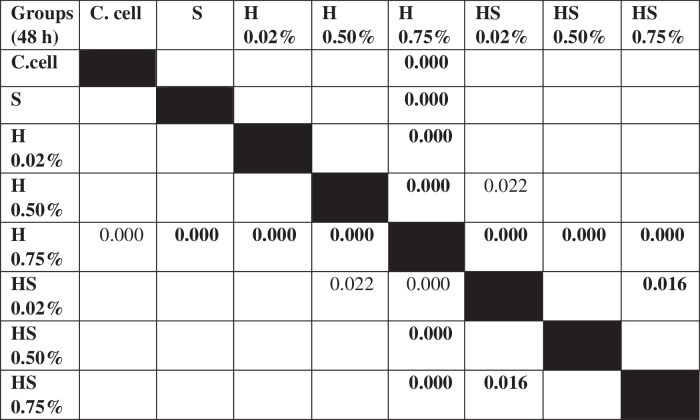
The *P* value of the Tukey HSD test for BHK-21 fibroblast cell migration between each study group (48 h).

## Discussion

In 2003, Miura et al. made a groundbreaking discovery when they identified a new type of multipotent and highly proliferative MSCs known as SHEDs. These dental mesenchymal cells were isolated from naturally exfoliated primary incisors of children aged 7 to 8 and derived from cranial neural crest cells (NCCs) [13]. Compared to other dental stem cells like periodontal ligament stem cells (PDLSCs) and DPSCs, SHEDs have been proven to be a population of highly proliferative and clonogenic cells [44]. One of the main biological properties of SHEDs that makes them ideal for dental tissue engineering is their ability to differentiate into osteo/odontoblasts. Over the last decade, various biologically active molecules and molecular pathways that influence this activity have been studied, shedding some light on osteogenic-induced differentiation [45].

Recent studies demonstrated the potential of SHED as a cell source for promoting osteogenesis in animal models without triggering immune rejection, although they may appear to be at odds with one another. However, more research involving longer follow-up times is required to ascertain which inductive combinations of SHED and biomaterials are most suitable for achieving the best outcomes [45]. The release of bioactive molecules from the SHED secretomes has a paracrine effect that is now being taken into account for regenerative medicine, but the mechanism of action of this kind of therapy is a crucial and frequently thought-about factor. The regenerative and protective qualities of various fractions of the secretome derived from SHED are being thoroughly tested and analyzed [25, 46–48]. In the current research, Human SHED-derived secretome was combined with Yemeni Sidr Honey to explore its impact on the viability and migration of osteoblast and fibroblast cell cultures.

**Fig. 9:**
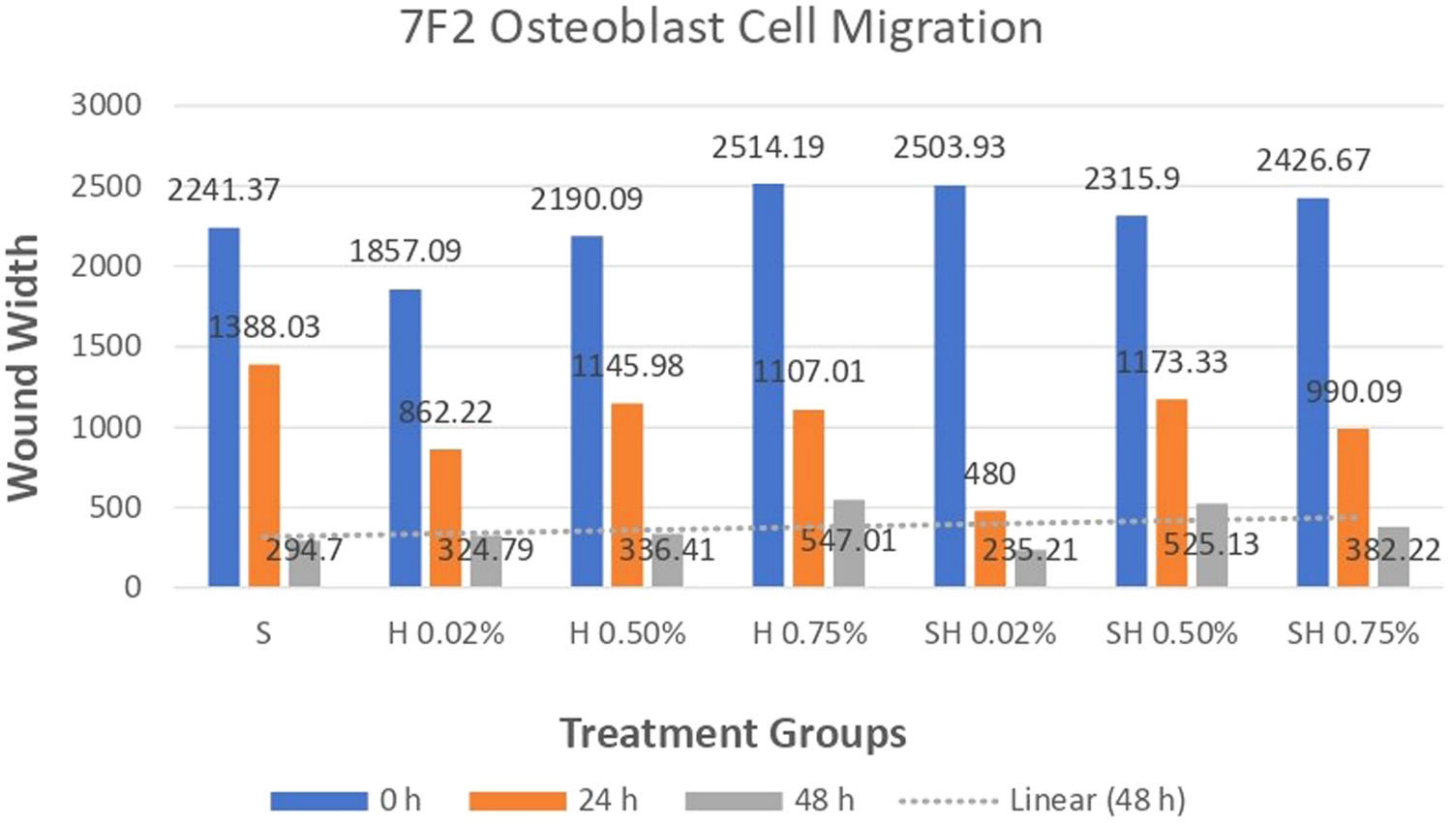
Cell Migration of 7F2 Osteoblast Cells.

**Fig. 10:**
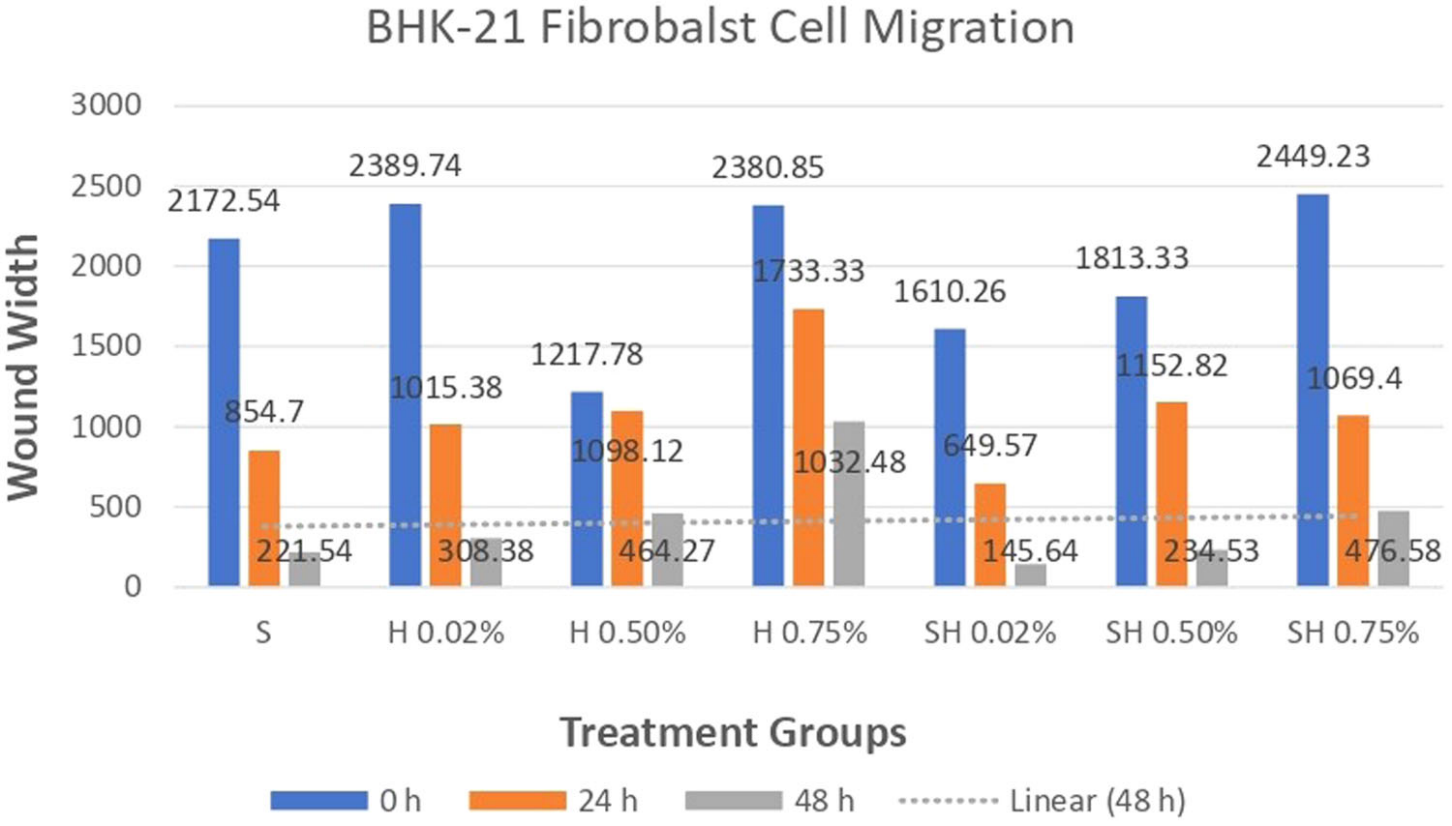
Cell Migration of BHK-21 Fibroblast Cells.

Human SHEDs release substances that have a strong capacity for regeneration [25, 49, 50]. Osteogenesis, which involves bone formation through osteoblasts, is directly related to bone repair [49]. Some Studies found that SHED-derived secretome stimulates osteogenesis by upregulating the expression of osteogenic genes and improving the migration and mineralization potential of stem cells. Osteoblastic differentiation is improved, osteochondral regeneration is enhanced, and bone defect healing is made possible by the secretome of SHEDs. In a study by Hiraki et al. SHED-derived conditioned medium (SHED-CM) was noticed to have a greater therapeutic potential for bone regeneration than the stem cells (SHEDs) [51]. In the present study, the findings demonstrated the power of SHED-derived secretome to promote cell viability of both osteoblasts and fibroblasts with cell viability percentages (90.70%,82.58%) respectively Figs. 1, 3. The measurement of cell viability was conducted via the MTT assay, a widely used colorimetric test for evaluating cytotoxicity and cell viability. This assessment primarily gauges cell viability by analyzing the mitochondrial performance of cells, which is determined by measuring the activation of mitochondrial enzymes. The result can be quantified by light absorbance at a specific wavelength. The amount of absorbent indicates the number of viable cells in the culture media. Moreover, a high capacity of cell migration was registered when using Scratch assay by measuring the length of the wound after treatment with SHED-derived Secretome in (0,24,48) hrs. Scratch length was measured in four sections of each image between the closest spots on both sides of the wound Figs. 5, 7. The more decreased the scratch length, the higher cell migration results. Scratch length was minimized after applying secretome for both osteoblasts (2241, 1388, 294.7) and fibroblast (2172, 854.7, 221) in (0,24,48) hrs. respectively Figs. 9, 10. This is similar to previous findings showing SHED-CM improved chondrocyte viability, which could be attributable to the availability of growth factors that increase proliferation and growth [52]. Ahangar et al. also demonstrated resembling outcomes wherein the injection of 100 μL of 20× Multipotent Adult Progenitor Cells Conditioned Media (MAPC-CM) into the wound margins resulted in improved rates of scratch closure, increased cellular proliferation, and improved angiogenesis [53]. Additionally, SHED-CM has also been found to greatly improve cell viability of human umbilical vein endothelial cells (HUVECs) in MTT assays and to speed HUVEC migration in wound healing and Boyden chamber assays. In a mouse Matrigel plug assay, the migrating number of primary endothelial cells was significantly higher in the plug containing SHED-CM or SHED suspension [54]. Moreover, SHED-derived exosomes were demonstrated to promote angiogenesis in human umbilical vein endothelial cells (HUVEC) under hyperglycemic circumstances, including enhanced cell proliferation, migration, tubular structure development, GATA2 gene expression, and CD31 protein expression which suggests that the use of SHED-exosomes may give a new therapy option for periodontal patients with diabetes mellitus [55].

Recently, much research has focused on utilizing honey for biomedical and wound healing purposes due to its advantageous properties [56]. The healing benefits of honey are attributed to its anti-inflammatory characteristics, which have been found to reduce the levels of pro-inflammatory cytokines. Honey has been shown to enhance the formation of new blood vessels, skin cell regeneration, and the creation of granulation tissue, as demonstrated by studies conducted in both laboratory and live animal settings [28]. Yemeni Sidr honey is well-known for its antibiotic, antibacterial, anti-inflammatory, antipyretic, and analgesic properties due to the abundance of minerals and elements it contains [29, 31]. Our research findings indicated that Yemeni Sidr Honey has the potential to enhance the viability and migration of osteoblast and fibroblast cells. However, lower concentrations of honey lead to higher cell viability and migration of both cells. Cell viability values among honey treatment groups with concentrations of (0.02%,0.50%,0.75%) were (87.82%,82.49%,74.56%) for osteoblasts Fig. 1 and (80.16%,77.16%, 70.57%) for fibroblasts respectively Fig. 3. While osteoblast and fibroblast cell migration were represented by the length of the wound, the registered values of wound length were (324.7, 336.4, 547) and (308.3, 464.2, 1032) respectively in 48 h (Tables 7, 11). This aligns with previous research showing that Tualang honey stimulates fibroblast cell proliferation at low concentrations (0.02%) but inhibits it at high concentrations [57]. Additionally, low concentrations of Pasture honey and Manuka honey have been found to activate phagocytes and stimulate lymphocyte proliferation which promotes wound healing [58]. Also, based on a study by Ranzato et al., it was demonstrated that various types of honey, including Acacia, Buckwheat, and Manuka, can speed up the healing process in a keratinocyte scratch assay, and enhance fibroblast migration in a transwell insert chemotaxis assay when used in low concentrations [59]. The increased proliferation may be due to honey’s carbohydrate content, which serves as a substrate for glycolysis [60]. Furthermore, According to Ebadi P et al., the Iranian propolis and honey samples efficiently impacted the migration, proliferation, and viability of human dermal fibroblast cells in a dose-dependent manner, however, no synergistic effects of the propolis and honey were identified [61]. It was also concluded that honey dilution can be beneficial in the healing of hypoxic wounds. This is because it reduces the formation of superoxide, while also creating an environment that supports cell proliferation, migration, and differentiation during the process of hypoxic wound healing. These findings underscore the potential benefits of using honey as a natural therapeutic substance for treating wounds in hypoxic conditions [62].

Based on the promising biological characteristics of honey, it has been combined with different (bio)polymers such as chitosan [63, 64], poly (vinyl alcohol) (PVA) [65], gelatin [17], and alginate [66] for tissue engineering and wound regeneration [67]. The current study proposes a combination of SHED-derived secretome and honey to promote bone healing and regeneration. According to our findings, the treatment groups of the combined secretome and honey (0.02%,0,50%.0.75%) had cell viability values of (95.90%, 94.25%, 90.83%) on osteoblasts and (86.53%, 85.80%, 82.94%) on fibroblasts respectively (Tables 2, 4). Furthermore, the results demonstrated that wound length varies between these groups on both cells osteoblast (235.2, 525.1, 382.2) and fibroblast (145.64, 234.53, 476.58) respectively in 48 h (Tables 7, 11). Accordingly, combining SHEDs Secretome and honey inhibits the formation of osteoclasts while favoring bone formation by osteoblasts and fibroblasts, thereby preserving and improving bone density and strength, ultimately leading to better bone healing and regeneration.

Considering all of the above, the findings of this research highlighted the efficacy of stem cell-derived secretome from human exfoliated deciduous teeth and Yemeni Sidr honey in promoting cell viability and migration for both osteoblasts and fibroblasts with due respect to some limitations related to the nature of in vitro settings research.

## Conclusion

The study concludes that combining SHED-derived secretome with Yemeni Sidr honey has the potential to enhance cell viability and migration in in-vitro settings. The application of these substances in a synergistic way has been shown to be more effective than their individual application. The effectiveness of the combined substances is directly proportional to the dose. However, it is essential to note that this is an in vitro study, which has certain limitations that need to be considered. One of the challenges is the difficulty of replicating the in vivo niche architecture, which includes cell-cell interactions and the accurate extracellular matrix milieu. These factors can potentially affect cell metabolism. Another limitation is the variation between cell culture conditions and human-like physiological media, which can influence cell signaling pathways. Additionally, the study faces constraints related to the short half-life of the isolated secretome and difficulties in maintaining cytokine activity post-administration in the context of stem cell-secretome-based therapy.

### Suggestions and future directions

To validate the findings of this study, additional in vitro experiments might be conducted with diverse secretome types and honey varieties. Furthermore, it is also imperative to carry out further in vivo animal research to thoroughly investigate the potential of combining the human exfoliated deciduous tooth stem cell-derived secretome with Yemeni Sider Honey in the context of regenerative medicine and tissue engineering. Such research is crucial before any clinical applications can be considered for human subjects. All in all, this research can be used as a starting point for further investigations to determine the possible clinical applications of the explored substance.

### Supplementary information

Supplementary Information

## Data Availability

All data supporting the findings of this study are available within the paper and its supplementary information,
