# Supplementary figures and images for: Combination of stem cell-derived secretome from human exfoliated deciduous teeth with Yemeni Sidr honey on cell viability and migration: an in vitro study
Source: BDJ Open. 2024 Mar 13;10:21. doi: 10.1038/s41405-024-00197-5 (PMC10937720; doi:10.1038/s41405-024-00197-5)

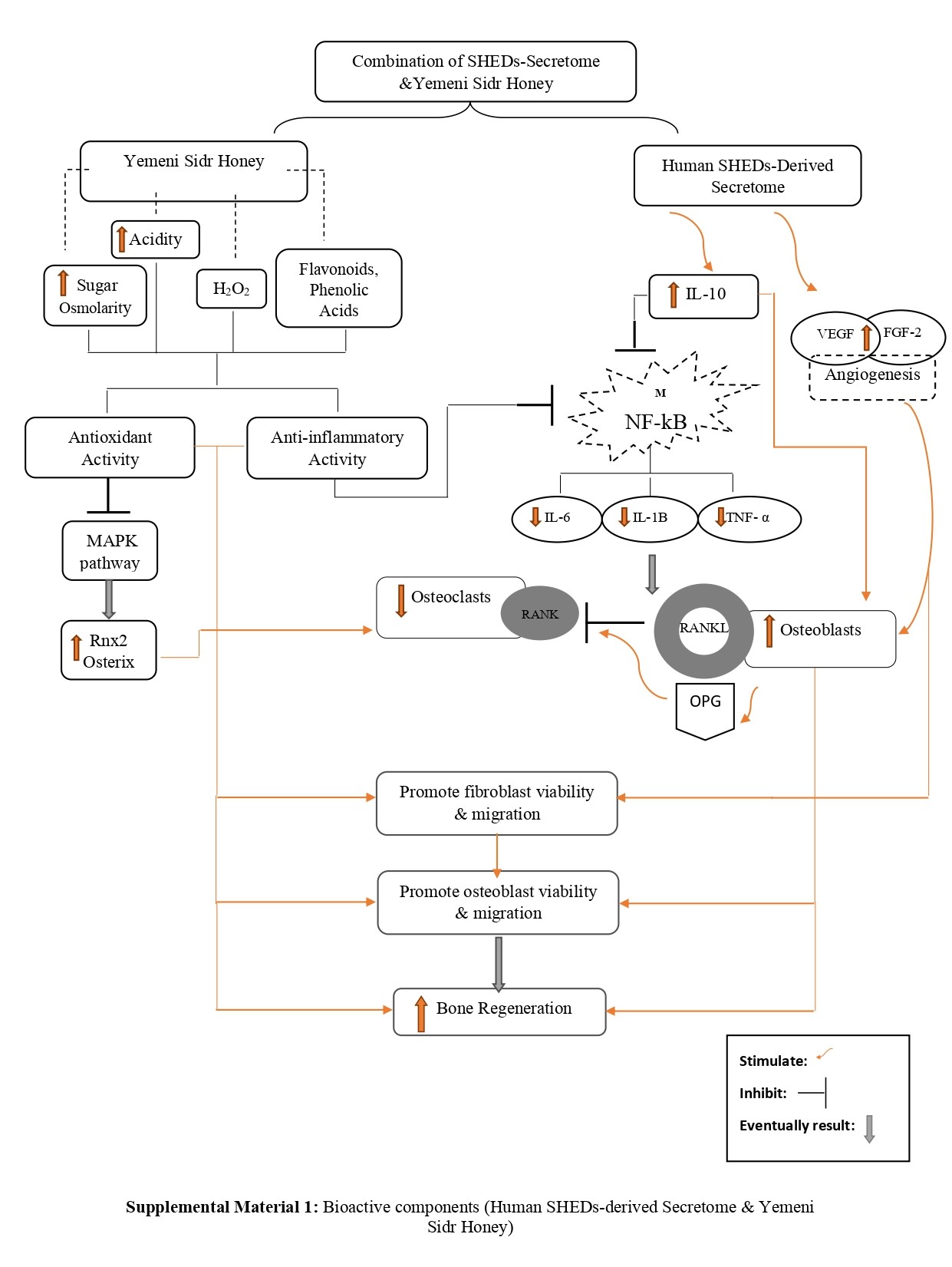

Supplement: Supplementary file 1 — Supplementary Information [file 41405_2024_197_MOESM1_ESM.jpg]
